# Moving from dementia risk disclosure to return of individual research results: a bioethics perspective

**DOI:** 10.1016/j.lanhl.2025.100723

**Published:** 2025-06-14

**Authors:** Bilal Irfan, Kelly Bakulski, Jonathan Reader, Annalise Rahman-Filipiak

**Affiliations:** Center for Bioethics, Harvard Medical School, Boston, MA 02115, USA; Center for Surgery and Public Health, Brigham and Women’s Hospital, Boston, MA, USA; Michigan Alzheimer’s Disease Center, Ann Arbor, MI, USA; Department of Neurology, University of Michigan Medical School, Ann Arbor, MI, USA; Department of Epidemiology, University of Michigan School of Public Health, Ann Arbor, MI, USA; Michigan Alzheimer’s Disease Center, Ann Arbor, MI, USA; Department of Epidemiology, University of Michigan School of Public Health, Ann Arbor, MI, USA; Michigan Alzheimer’s Disease Center, Ann Arbor, MI, USA; Department of Neurology, University of Michigan Medical School, Ann Arbor, MI, USA; Michigan Alzheimer’s Disease Center, Ann Arbor, MI, USA; Research Program on Cognition and Neuromodulation Based Interventions, Department of Psychiatry, University of Michigan, Ann Arbor, MI, USA

In dementia prevention research, language use is not a cosmetic choice but a constructive act that directs both the participants’ expectations and the investigators’ duties. Describing feedback on patient-level or participant-level genetic, imaging, fluid, or behavioural indicators as risk disclosure denotes a paternalistic frame in which researchers own these data and decide whether to unveil a potentially stigmatising secret. Recasting the same activity as the return of individual research results (RoR) recognises that the data originate with the participants, who therefore hold a prima facie claim to receive them. This shift in terminology is ethically and scientifically preferable and comports with emerging perspectives on ageing and brain health.

A complex systems analysis of functional ageing shows that cognition in late life is an emergent condition arising from dynamic interactions among physical, psychological, cognitive, and social domains; isolating a single biomarker as a stand-alone predictor for functional ageing overstates its causal weight, as it can only contribute a small, although independent, prognostic value.^1^ The recent Personal View in *The Lancet Healthy Longevity* by Kok and colleagues rejects this reductionism in favour of network thinking.^1^ Simultaneously, the *Lancet Healthy Longevity* Editorial on the life course approach urges prevention strategies that begin early and are sustained across decades.^2^ A narrow framing of risk obscures the broader informational ecology necessary for resilience and could leave the participants with an isolated probabilistic label, whereas RoR recasts result sharing as a bidirectional dialogue in which every data point is interpreted alongside the participant’s broader life course and medical context.

The 2016 guidelines from the Council for International Organizations of Medical Sciences, prepared in collaboration with WHO, presume a duty to offer clinically or personally significant findings generated by research, providing analytical validity and an appropriate pathway for counselling.^3^ This duty is echoed in the Participant Bill of Rights created by the International Society to Advance Alzheimer’s Research and Treatment, which identifies result sharing as a fundamental entitlement.^4^ The US National Academies, after a comprehensive review of benefits, harms, and costs, reached the same conclusion, proposing a deliberative framework that balances scientific quality, participant preferences, and actionability while treating result sharing as the default manifestation of respect for participants.^5^ Such documents offer a starting point to abandon the vocabulary of secrecy and instead adopt bidirectional information flows that foster trust, reciprocity, and collective scientific progress.

In 2023, Largent and colleagues synthesised previous empirical work into a five-step disclosure pathway for amyloid and related biomarkers;^6^ their guidance conforms with that of multiple studies showing that structured counselling, confirmatory testing, and follow-up can yield high participant understanding and satisfaction without clinically significant increases in anxiety or depression.^7,8^ Largent and colleagues also suggested that many recipients used the information to adjust health behaviours or initiate family planning discussions,^6^ outcomes that could be unattainable under an information withholding model. These recommendations suggest that the ethical risk lies not in sharing results with the participants but in sharing them poorly; RoR frameworks prioritise process quality, rather than paternalistic restraint, as the central safeguard.

Terminology also shapes justice. RoR conveys reciprocity, acknowledging that the communities that supply biological samples and longitudinal data–often those bearing the greatest dementia burden–deserve concrete informational dividends. Withholding results fractures the feedback loops that maintain system-level resilience,^1^ whereas RoR enables participants and their families to adjust behaviours, environments, and expectations in synchrony with emerging evidence.

Ethicists working on complex biomarker studies have likewise argued that participant partnership, not secrecy, is the proper response to the growing precision and complexity of dementia-related metrics.^9^ Participant partnership includes transparency about personal results but also about the limitations and experimental nature of the tests themselves. For example, RoR should highlight that amyloid cutoffs are potentially inappropriate for some individuals as standard amyloid-positive thresholds might underdetect pathology in some populations.^10^

Regulatory clarity is an additional dividend. Labelling feedback as disclosure blurs the boundary between research and clinical care. By contrast, RoR frames result sharing as a positive act of stewardship and encourages protocols that begin with consent-based preference elicitation proceed through multilayered education and culminate in facilitated links to medical or community services. Studies adopting such designs have reported high participant satisfaction and perceived utility without evidence of psychological harm, supporting their scalability in large cohorts.^7–9^

Language is never entirely neutral. Words carry power. Continuing to use the term “risk disclosure” perpetuates an outdated model in which investigators control the flow of data, while participants passively accept or reject an anxiety-laden label. Embracing RoR affirms participant ownership, aligns with complex systems and life course science, and is in line with empirical evidence that shows that carefully mediated information can be shared safely. Therefore, we urge dementia investigators, research ethics committees, and journal editors to adopt RoR terminology and to embed their practices in culturally responsive, person-centred frameworks. Such a shift will enhance trust, foster equitable engagement, and accelerate the translation of dementia research into meaningful public health benefits for diverse populations.
